# Growing knowledge impact of gene-editing technology on public acceptance: a longitudinal analysis in Japan

**DOI:** 10.1080/21645698.2024.2435709

**Published:** 2024-12-06

**Authors:** Atsushi Sato, Daiki Watanabe, Yoko Saito

**Affiliations:** a Development Strategy Center, National Agriculture and Food Research Organization, Minato-ku, Tokyo, Japan; b Research Faculty of Agriculture, Hokkaido University, Sapporo, Hokkaido, Japan

**Keywords:** Genome editing technology, Japan, knowledge impact, public acceptance, technology diffusion

## Abstract

Genome editing (GE) technology holds significant promise for advancements in crop development and medical applications. However, public acceptance of GE in Japan remains uncertain. This study aimed to examine how knowledge impacts public acceptance of GE technology, focusing on differences across diffusion stages and application purposes. Using ordinary least squares regression on repeated survey data collected from 2018 to 2023 in Japan (*n* = 6,234), we investigated the influence of knowledge on support for GE in consumer benefits, producer benefits, and medical technology. Our findings revealed that the effect of knowledge on technology acceptance has strengthened over time. Consumers with greater knowledge of GE are increasingly supportive of its advancement, emphasizing the growing importance of information as the technology becomes more widespread. This research highlights the role of transparent policy discussions in fostering public trust and support, thereby promoting the successful integration of new technologies into society.

## 1. Introduction

### 1.1. Background

The world currently faces a significant threat of food insecurity.^1^ In 2023, nearly 282 million people experienced acute food insecurity, requiring urgent food and livelihood assistance.^2^ The Food and Agriculture Organization of the United Nations (FAO) projects that global agricultural output will need to increase substantially to meet future demand,^3^ especially with rising demand for animal-based foods.^4^ However, global environmental changes have destabilized food supplies. Climate change and extreme weather events are increasingly common, negatively impacting agricultural productivity.^5^ Climate variability has exacerbated food insecurity in regions already struggling with hunger and malnutrition,^6^ highlighting the urgent need for resilient crop varieties to secure future harvests.^7^

Genome editing (GE) technology presents a potential solution to address growing food demands and diverse food preferences.^8^ Many biotechnologists advocate that GE is more effective than conventional methods for developing crop varieties with superior agronomic performance and quality.^9^ Among recent genetic advances, technologies such as Clustered Regularly Interspaced Short Palindromic Repeats (CRISPR) are being applied in plant and animal breeding. GE allows for precise genome modifications than traditional breeding methods and can reduce both the breeding period and development costs for new varieties.^10^ GE technology is employed to develop a range of agricultural products, such as drought-tolerant maize^11^ and allergen-free chicken eggs.^12^ Beyond agriculture, GE technology is also widely applied in animal and human research.^13^ In medicine, GE has been instrumental in understanding genetic diseases and developing treatments for conditions such as Parkinson’s Disease^14^ and AIDS.^15^

### 1.2. Development of Genome-Edited Products in Japan

Product development using GE technology has progressed in Japan, where this study was conducted, with several items already available on the market. In 2021, Sanatech Seed introduced the world’s first CRISPR-edited tomato, which provides health benefits, including blood pressure reduction, in the Japanese market.^16^ Sales of two CRISPR-edited fish, tiger puffer and red sea bream, which require less feed to reach market size, have also begun.^17^ The tiger puffer’s growth rate is enhanced by disrupting the leptin receptor gene, while the red sea bream’s growth is boosted by disabling the myostatin protein.

Although the implementation of GE products in Japan has been successful so far,^18^ genetically modified (GM) crops faced strong opposition from consumer groups in the early 2000s. To date, Japan has approved 156 GM crop varieties for cultivation, distribution, and import.^19^ However, the only GM crop cultivated commercially is an ornamental blue rose. While GM crops such as soybean, rapeseed, corn, and cotton are imported in large quantities for feed, edible oil, and as raw materials for processed foods, they are not grown commercially for food production within Japan. GE products, though present in the market, are still not widespread. Previous studies have indicated that consumers generally perceive GE technology more positively than GM techniques, though less favorably than conventional methods.^20–24^ Therefore, it is crucial to identify the factors that limit consumer acceptance of GE products, even after market introduction.

### 1.3. Knowledge of Gene Editing Technology

Many studies have identified factors influencing consumer acceptance of new technologies.^25–31^ Knowledge and information play a key role in shaping consumer attitudes^32–36^ toward GM technology. These discussions typically cover: (1) positive or negative information about the technology,^32^ (2) perceived benefits and risks,^33^ (3) environmental and health benefits,^34^ (4) familiarity with the technology,^35^ and (5) both subjective and objective knowledge of GM technology.^36^ Numerous studies have examined how specific types of knowledge and information content influence consumer evaluations, indicating that knowledge generally promotes greater acceptance of new technology.^21,23,24,37^ For example, Shew et al.^21^ found that information on biotechnology for both GM and GE foods can raise willingness to pay to levels comparable with conventional foods. Ortega et al.^38^ also found that knowledge of GE technology is a significant factor in shaping consumer attitudes, and Son & Lim^23^ highlighted that consumers’ scientific knowledge is closely related to their acceptance of GE technology, underscoring the importance of relevant information for diffusion.

Most prior research has examined acceptance at a single stage of the diffusion process. However, as Frewer^29^ observed, the factors influencing acceptance may change throughout the diffusion process. This suggests that public acceptance and the factors affecting it can vary across different stages of technology diffusion. Additionally, recent food-related incidents may significantly influence public perceptions of new technologies. Therefore, the impact of information and knowledge may differ depending on the technology’s diffusion stage and relevant social events.^29,39^ For instance, Läpple et al.^40^ found that factors influencing the adoption of organic farming differ across diffusion stages. Similarly, Waarts et al.^41^ showed that factors influencing new software adoption vary between early and late adopters. In the case of nanotechnology, van Giesen et al.^39^ noted that, in early stages of technological innovation, people often respond emotionally rather than cognitively when making adoption decisions. Therefore, factors influencing adoption differ across diffusion stages, and knowledge – a cognitive factor – may become increasingly important as technology becomes more widely disseminated.

### 1.4. Different Perceptions for Different Application Purposes

GE technologies are applied to a range of products, from agricultural and marine items to medical purposes. However, consumer acceptance is often evaluated based on specific products,^20,37,42–44^ such as rice,^21^ potatoes,^22^ soybean oil,^23^ and orange juice.^24^ Some studies have compared acceptance across multiple products, such as rice and pork,^38^ milk, apples and potatoes,^45^ or soybean oil and apples.^46^ Kato-Nitta et al.^37^ found that GE applications in vegetables were better accepted than those involving livestock. Since GE technology can be applied to a wide variety of products, consumer evaluations for specific items may not be relevant to others.

Studies on consumer acceptance also consider the purpose of GE applications.^22,47^ For example, Muringai et al.^22^ conducted choice experiments with Canadian consumers to evaluate GM/GE potatoes and found that consumers were more willing to pay for health benefits than for environmental benefits. In Norway, most consumers supported GE applications in agriculture and aquaculture^47^ but became negative if the technology altered the appearance of plants or animals or increased livestock productivity. Medical applications of GE technology have also been evaluated by purpose.^13,48–52^ Busch et al.^48^ found that using GE to cure diseases is more acceptable than applying it to agricultural or food products. Gaskell et al.^50^ identified diverse public responses to medical applications, noting that technologies aimed at enhancing functions, such as memory and learning ability, were less acceptable than those intended to treat diseases. McCaughey et al.^52^ showed that the public generally supports GE for curing life-threatening diseases but not for genetic enhancement. A similar trend was found in Australia.^13^ Japanese citizens also strongly oppose GE use for enhancement purposes, though clinical use is acceptable.^49^ Applications aimed at disease prevention and treatment receive the most support, while changes to non-life-threatening traits receive the least.^51^

### 1.5. Objectives of the Study

This study has two main objectives. First, it aims to empirically demonstrate how the impact of knowledge changes across different diffusion stages by using survey data collected over several years. The survey period includes food-related incidents such as the COVID-19 pandemic^53^ and the Russia-Ukraine conflict,^54^ which may have influenced public attitudes. We also investigate whether public acceptance varies by the intended applications of GE technology, covering a wide range of items and application purposes, including agricultural products and medical technologies. If, as van Giesen et al.^39^ suggest, the impact of knowledge shifts depending on the diffusion stage, then distinct policy approaches may be needed to effectively integrate the technology into society.

Second, we hypothesize that the impact of knowledge on acceptance may also differ by application purpose. In other words, knowledge may have a stronger effect on acceptance for specific purposes, such as agricultural productivity enhancement. If our hypothesis is supported, policy interventions – such as public information campaigns or outreach activities – could be particularly effective during the later stages of new technology dissemination. To the best of our knowledge, no previous research on GE technology has specifically investigated consumer responses with a focus on how the impact of knowledge changes over time.

The remainder of this paper is organized as follows: Section 2 discusses our online survey methodology, a factor analysis of the items, and an analysis of participants’ awareness and perceptions of GE technology. Section 3 describes the estimation model and data collection procedures. Section 4 presents the results of the regression analysis, emphasizing the effectiveness of information across different diffusion stages. Finally, Section 5 discusses effective policies for integrating new technology into society.

## 2. Survey and Data

### 2.1. Online Survey

The survey was conducted five times between January 2018 and February 2023 (Table 1). For each survey, participants were independently selected from a pre-registered sample provided by the online survey company, Macromill, Inc. Table 1 displays the number of respondents in each survey. Since the survey was administered by a research firm, the sample data were anonymized, and we were only given access to respondent ID numbers, which are individually assigned upon registration in Macromill’s pre-registered pool.

**Table 1.** Sample characteristics.

Survey period	Jan–Feb 2018	Jan–Feb 2019	March 2021	Feb 2022	Feb 2023
Questionnaire title	Food questions	Food questions	Survey on personal information	Survey on personal information	Survey on personal information
Gender					
Male	620 (50%)	775 (50%)	630 (50%)	539 (50%)	555 (50%)
Female	620 (50%)	775 (50%)	630 (50%)	539 (50%)	551 (50%)
Age					
20s	248 (20%)	310 (20%)	252 (20%)	213 (20%)	221 (20%)
30s	248 (20%)	310 (20%)	252 (20%)	217 (20%)	221 (20%)
40s	248 (20%)	310 (20%)	252 (20%)	214 (20%)	221 (20%)
50s	248 (20%)	310 (20%)	252 (20%)	218 (20%)	220 (20%)
60s and above	248 (20%)	310 (20%)	252 (20%)	216 (20%)	223 (20%)
Education Level					
Junior high school graduate	25 (2.0%)	35 (2.3%)	36 (2.9%)	29 (2.7%)	32 (2.9%)
High school graduate	335 (27%)	414 (27%)	315 (25%)	278 (26%)	285 (26%)
Junior college or vocational or special school graduate	281 (23%)	369 (24%)	301 (24%)	256 (24%)	252 (23%)
Dropped out of university (Some university classes)	26 (2.1%)	32 (2.1%)	26 (2.1%)	11 (1.0%)	22 (2.0%)
University or college graduate	501(40%)	635(41%)	527(42%)	448(42%)	453(41%)
Graduate school graduate	70(5.7%)	60(3.9%)	63(5.0%)	54(5.0%)	60(5.4%)
Others	2 (0.2%)	5 (0.3%)	2 (0.2%)	2 (0.2%)	2 (0.2%)
Children					
No children	558 (45%)	695 (45%)	580 (46%)	526 (49%)	546 (49%)
Has children	682 (55%)	855 (55%)	680 (54%)	552 (51%)	560 (51%)
Awareness					
Aware	513(41%)	805(52%)	684(54%)	574(53%)	548(50%)
Not aware	727(59%)	745(48%)	576(46%)	504(47%)	558(50%)
	*n* = 1,240	*n*= 1,550	*n* = 1,260	*n* = 1,078	*n* = 1,106

Respondents were recruited based on a questionnaire sent by the survey company or posted on the company’s website, and recruitment continued until the desired sample size was reached. We requested an equal number of male and female respondents within the age range of 20–60, with no additional sampling criteria such as region or occupation. This approach resulted in nearly identical proportions of respondents by gender and age across surveys. Since the survey was conducted online, and we selected respondents based on balanced age and gender criteria, the sample may not fully represent the Japanese demographic structure. However, this allocation enables us to examine the specific effects of socio-economic variables, such as age and gender, on the social acceptance of GE technology. This method of equally allocating by age and gender has also been used in previous studies on consumer acceptance.^42,55,56^

The GE technology examined in this study raises ethical and sensitive issues, potentially leading to social desirability bias, where respondents may provide socially desirable answers rather than their true opinions. This could result in responses that differ from actual beliefs or behaviors. To help mitigate this bias, a web-based survey was selected to ensure respondent anonymity and avoid face-to-face interaction with researchers. Additionally, to reduce opinion bias during recruitment from the pre-registered sample, specific terms such as “GE technology” were avoided in the questionnaire title. Instead, more general terms such as “survey on food” and “survey on personal information” were used. Each year’s sample excluded respondents who had participated in the previous year.

Respondents’ demographic characteristics are presented in Table 1. The proportion of respondents with children ranged from 51% to 55%, while years of education varied slightly from year to year. Socioeconomic characteristics remained consistent across all survey years. A chi-square test was conducted to compare the distribution of demographic factors (gender, age, education level, and parental status) and showed no statistically significant differences between survey groups, indicating that the sample over these five years was nearly homogeneous in terms of sociodemographic attributes. Respondents also answered seven questions on their subjective knowledge of GE technology (*know_GE*) and 12 questions on their interest in science and technology (*tec*), each on a Likert scale. Details are provided in the Supplementary Materials (Tables A1 and A2).

### 2.2. Awareness and Acceptance

Table 1 shows the level of awareness across the five surveys conducted since 2018. “Being aware” corresponds to respondents who are “very knowledgeable,” “have some information,” or “have heard of it,” while “Not being aware” includes respondents who know “nothing at all” or “not much,” based on responses to seven questions (see Supplementary Table A1). The 2018 survey had the lowest awareness level, with approximately 59% of respondents reporting low awareness. Awareness increased between 2018 and 2019; however, in 2022 and 2023, the most recent surveys, awareness declined again, with approximately 50% of respondents indicating low awareness. The rate of awareness has fluctuated rather than steadily increasing and has shown a recent decline.

### 2.3. Factor Analysis on Different Items

#### 2.3.1. Factor Analysis

GE technology serves various purposes, and consumers’ intentions to promote GE products are expected to vary depending on the product’s purpose. In this survey, respondents were asked about their “intention to promote” 24 products currently in development or anticipated in Japan. Responses were rated on a 5-point Likert scale (1 = not advisable to promote, 2 = somewhat not advisable to promote, 3 = no opinion/neutral, 4 = somewhat advisable to promote, and 5 = highly advisable to promote). We employed factor analysis to classify these items into narrower groups, which we applied to the surveys conducted in later years as well. All analyses were conducted using the psych package^57^ in the R statistical software.^58^

The results of the factor analysis are presented in Table 2. The number of factors were determined using the Kaiser criterion,^59^ where eigenvalues greater than 1.0 indicate significant factors, and by assessing the interpretability of each factor. Studies across various fields have commonly employed the eigenvalue > 1.0 criterion for factor selection.^60–62^ Three factors with eigenvalues greater than 1.0 were identified, and factor loadings greater than 0.4 are highlighted in bold and underlined.

**Table 2.** Results of factor analysis.

Items	Factors		
	Producer benefits	Medical technology	Consumer benefits
Tomatoes and melons with high sugar content	0.010	−0.032	**0.720**
Potatoes that do not produce solanine	0.071	−0.027	**0.679**
Decaffeinated tea	0.079	−0.072	**0.642**
Apples that do not turn brown	0.318	−0.109	**0.468**
Delicious rice	−0.057	−0.007	**0.843**
Feed rice that is high in amino acids	0.243	0.018	**0.580**
Allergy-free buckwheat	−0.087	0.077	**0.817**
Vegetables effective in treating dementia	−0.091	0.231	**0.702**
Rice effective in treating hay fever	−0.001	0.151	**0.648**
Flowers with unusual colors and shapes	**0.519**	−0.088	0.100
Apples with fast breeding speed	**0.616**	−0.046	0.170
Corn, forage, and rapeseed with herbicide resistance	**0.945**	0.087	−0.137
Wheat with herbicide resistance	**0.983**	0.098	−0.179
Oilseed soybeans with resistance to pests	**0.890**	0.097	−0.103
Rice and papaya with disease resistance	**0.788**	0.114	−0.014
Tuna with high aquaculture efficiency	**0.538**	0.091	0.225
Pigs with increased meat production	**0.665**	−0.014	0.110
Cattle without horns	**0.625**	−0.142	0.009
Treatment of muscular dystrophy	0.021	**0.852**	−0.058
Prevention of AIDS onset in patients	0.029	**0.866**	0.011
Treatment of Parkinson’s disease	−0.035	**0.918**	−0.001
Treatment of cancer	−0.022	**0.808**	0.073
Prevention of hereditary diseases	0.017	**0.821**	0.066
Recovery from liver disease	0.045	**0.894**	−0.002
SS loadings	5.394	4.782	4.631
Cumulative Variance	0.225	0.424	0.617
Proportion Explained	0.364	0.323	0.313

Factor loadings greater than 0.4 are shown in bold.

**Figure 1.**
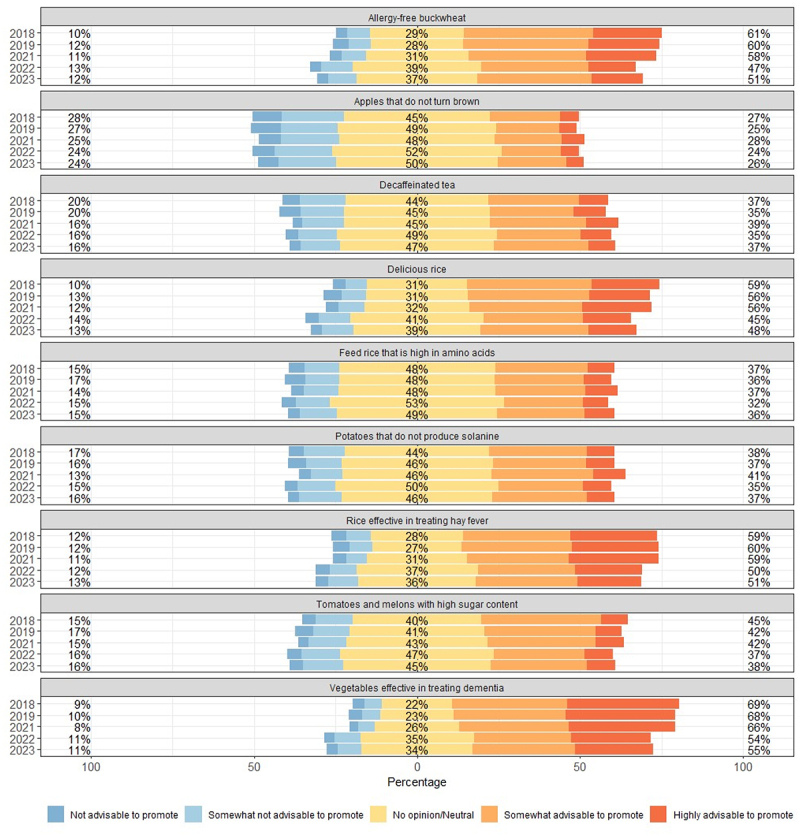
Evaluation results for consumer benefits.

**Figure 2.**
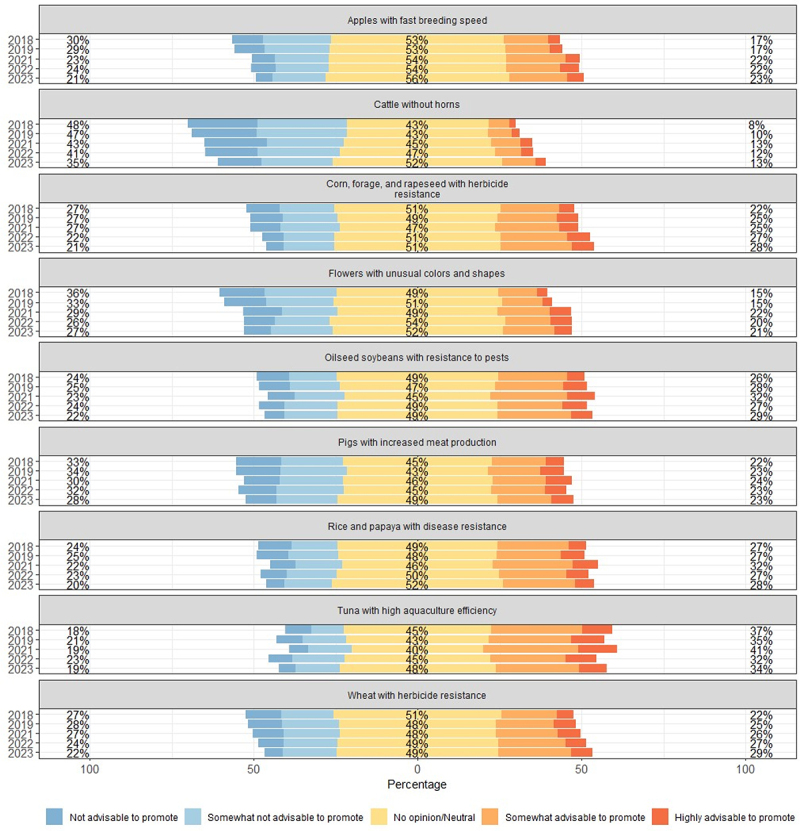
Evaluation results for producer benefits.

**Figure 3.**
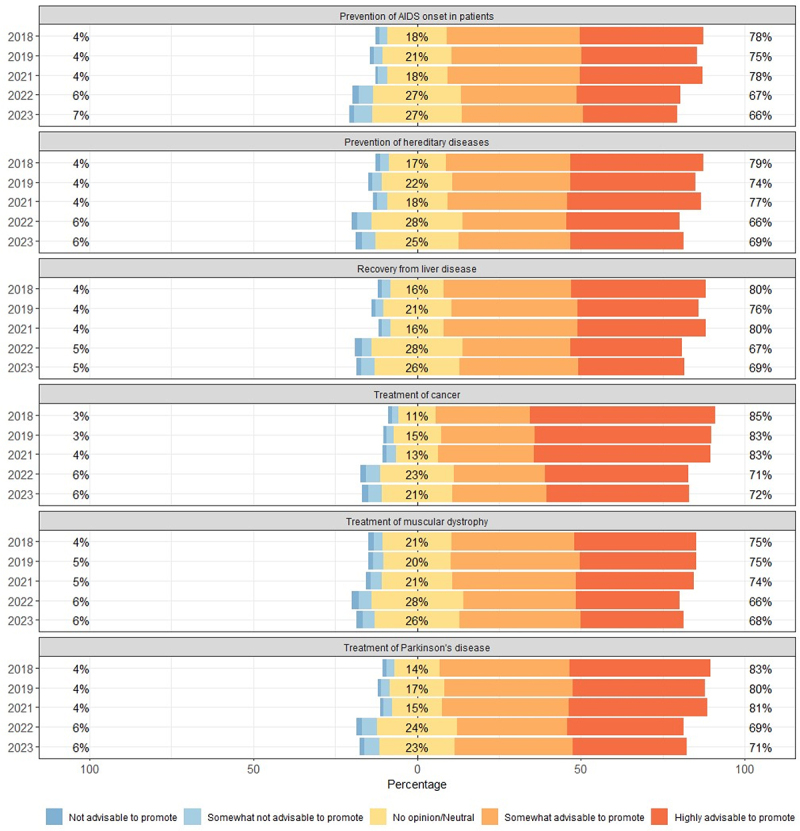
Evaluation results for medical technology.

Factor 1 is labeled “Producer Benefits (PROD)” and includes items such as herbicide-resistant and disease-resistant agricultural products (e.g., corn, forage, wheat) and products aimed at increasing production efficiency, such as “high aquaculture efficiency tuna” and “pigs with enhanced meat yield.” Factor 2 has a high loading for items such as “treatment for muscular dystrophy,” “cancer treatment,” and “Parkinson’s disease treatment,” and is named “Medical Technology (MED).” Factor 3 groups items focused on enhancing consumer appeal, such as improving the taste of “high-sugar-content tomatoes and melons” and “delicious rice,” as well as items improving functionality such as “decaffeinated tea” and “allergy-free buckwheat.” This factor is named “Consumer Benefits (CONS).” The detailed results for each item are shown in Figs. 1–3.

#### 2.3.2. Acceptance of Three Categories

The average “intention to promote” score for each category (CONS, PROD, and MED) is employed to evaluate consumer perceptions of GE technology, serving as the dependent variable in the estimation model presented in the following section. For each category, scores were averaged: CONS included nine items, while PROD and MED each represent scores specific to their respective items. Additional details are provided in Supplementary Table A3.

Descriptive statistics are presented in Table 3. A one-way analysis of variance (ANOVA) was conducted for all survey years to compare mean scores across categories. The null hypothesis, stating that “the population means for the intention to promote technology are similar across the three categories,” was rejected (*p* < .01), indicating statistically significant differences in mean scores among at least one of the three categories. The Bonferroni method for multiple comparisons was used to further clarify these relationships. Results showed that the MED category had the highest acceptance rate, followed by CONS and then PROD (*p* < .01). This suggests respondents hold a more favorable view of medical applications (MED) of GE technology than applications to agricultural products (CONS and PROD). This finding aligns with Connor & Siegrist’s^63^ research, which found that respondents in Switzerland were more skeptical about genetic engineering in food compared to medical applications. It also corresponds with prior studies indicating strong public support for medical GE applications in Japan.^64^

**Table 3.** Descriptive statistics for variables.

Variable	Variable definition	2018	2019	2021	2022	2023
		*n* = 1,240	*n* = 1,550	*n* = 1,260	*n* = 1,078	*n* = 1,106
**Dependent variables**						
*CONS*	Average score of the 9 items in the CONS category	3.44 (0.75)	3.41 (0.79)	3.46 (0.73)	3.33 (0.78)	3.36 (0.76)
*MED*	Average score of the 6 items in the MED category	4.18 (0.78)	4.12 (0.79)	4.15 (0.76)	3.95 (0.89)	3.96 (0.88)
*PROD*	Average score of the 9 items in the PROD category	2.85 (0.77)	2.88 (0.81)	2.96 (0.76)	2.95 (0.79)	3.00 (0.73)
**Independent variables**						
*d_fem*	1 for female and 0 for male	620 (50%)	775 (50%)	630 (50%)	539 (50%)	555 (50%)
*age*	Age in years	44 (14)	45 (14)	45 (14)	45 (14)	44 (14)
*d_child*	1 if the respondent has children, 0 otherwise	682 (55%)	855 (55%)	680 (54%)	552 (51%)	560 (51%)
*d_edu*	1 if university/college graduate or postgraduate, 0 otherwise	571 (46%)	695 (45%)	580 (46%)	502 (47%)	513 (46%)
*tec*	Awareness of indicated technologies^a^	3.70 (0.63)	3.73 (0.63)	3.84 (0.59)	3.80 (0.71)	3.79 (0.69)
*know_GE*	Knowledge about gene editing^b^	2.04 (0.94)	2.21 (0.95)	2.15 (0.91)	2.10 (0.93)	2.14 (0.93)

Note 1: Continuous variables are presented as mean (standard deviation). Dummy variables are expressed as the number of cases with a value of 1%).

Note 2: ^a^The average score of responses to 12 questions regarding the intention to promote science and technology development (Appendix: Table A2).

Note 3: ^b^The average score of responses to 7 questions regarding knowledge of genome editing technology (Appendix: Table A1).

These differences in acceptance across categories may be attributed to the varying benefits perceived by respondents. For instance, medical applications of GE often provide direct benefits, such as disease treatment and prevention, that can significantly enhance patient quality of life. Thus, respondents tend to support GE applications in medical contexts more enthusiastically, recognizing the clear health benefits.

The response to GE technology in agricultural products appears to vary between CONS and PROD, likely due to differences in perceived benefits. While both categories relate to agriculture, the direct consumer benefits in CONS (e.g., improved food quality) may be more highly valued than the indirect benefits in PROD, which focus on social issues such as productivity and animal welfare. Although PROD offers significant advantages to producers, such as increased productivity in agriculture and fisheries, these benefits may be less apparent to consumers. Therefore, the ambiguity of the benefits in PROD may contribute to the varied responses among categories. The limitations of the survey format did not allow detailed explanations of each application, so responses were likely based on intuitive perceptions.

We compared the results across survey years. Among the three categories, PROD had the lowest initial acceptance, with a mean score of 2.85 in 2018. Initially viewed negatively, the “intention to promote” PROD has gradually increased over time. In the most recent survey, conducted in 2023, the average score reached 3.00, suggesting a shift from a negative to a neutral perception. This trend may reflect increasing public concern about food security due to recent events such as food-related incidents, COVID-19, and the Russia – Ukraine conflict, which have heightened awareness of the benefits of increased agricultural and fishery productivity associated with the PROD category.

Conversely, CONS and MED scores were relatively high from the beginning of the survey, though they fluctuated slightly year by year. CONS scores ranged between 3 (neutral) and 4 (should be promoted), while MED remained around 4 (should be promoted). However, public acceptance for both CONS and MED declined slightly by the 2023 survey.

### 2.4. Awareness and Impression

**Figure 4.**
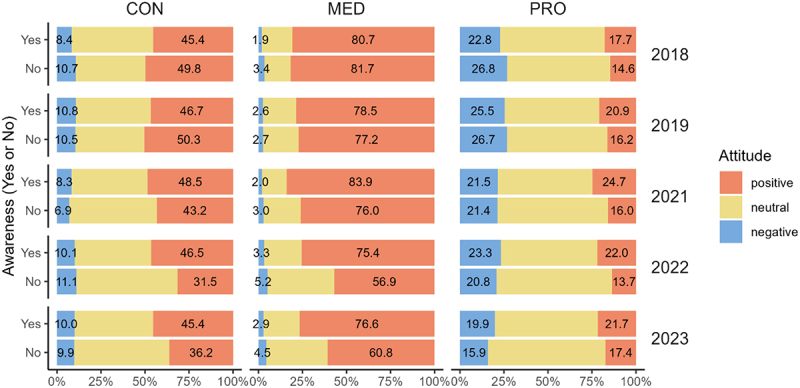
Awareness and attitudes toward gene editing technology.

To understand the impact of information on perception, we cross-tabulated respondents’ “awareness” of GE technology (No/Yes) with their “impression” (positive/neutral/negative) (Fig. 4). This figure shows the distribution of attitudes among those who were aware (Yes) versus unaware (No) of GE technology. Positive impressions were generally higher among respondents aware of GE technology, with some exceptions in CONS during 2018 and 2019. Overall, awareness tends to increase positive attitudes, especially in the later stages of technology diffusion, as observed in the 2022 survey. This suggests that the impact of information varies by the stage of technology diffusion.^29,39^

## 3. Methods

### 3.1. Estimation Model

This study uses an Ordinary Least Squares (OLS) regression model to identify factors influencing the “intention to promote” GE technology. Survey data from all five years were pooled and analyzed across three different categories. The estimation model is as follows, with the dependent variable being the intention to promote GE technology by respondent *i* in each of the three categories *j*.

$$Y_{j,i}=\beta_{0}+\underset{\mathrm{year}}{\sum }\beta_{d\_\mathrm{year}}\cdot d\_\mathrm{yea}r_{i}$$

$$+\beta_{\mathrm{know}\_\mathrm{GE}}\cdot \mathrm{know}\_GE_{i}$$

$$+\underset{\mathrm{year}}{\sum }\beta_{d\_\mathrm{year}\cdot \mathrm{knowGE}}\cdot d\_\mathrm{year}\cdot \mathrm{know}\_GE_{i}$$

$$+\beta_{\mathrm{socio}\_\mathrm{char}}\cdot X_{i}+\varepsilon_{i}$$

$$j=\left\{\mathrm{CONS},\mathrm{PROD},\mathrm{MED}\right\},$$

year-specific dummy variables (d_year = 2019, 2021, 2022, 2023) capture changes in evaluations across the survey period, with 2018 serving as the baseline. This period (2018–2023) includes significant events, such as the COVID-19 pandemic and the Russian-Ukraine conflict, which have influenced public perspectives on technology in China.^65^ We investigated whether similar shifts in valuation are observed among Japanese consumers over this period.

To address potential heteroskedasticity, we utilized Heteroskedasticity-Consistent Standard Errors calculated using the sandwich package^66,67^ in R. This approach ensures that our standard errors remain robust against violations of the homoskedasticity assumption, thereby providing more reliable statistical inferences. Additionally, to assess collinearity, we calculated the Generalized Variance Inflation Factor (GVIF) using the car package^68^ in R. The results showed that GVIF values for all variables were approximately 1.2, which satisfies the condition 1.0 < GVIF < 5.0. Therefore, we retained all variables in the model without removing any that were associated with multicollinearity.

### 3.2. Data

The interaction term between the year dummy and knowledge (*d_year*⋅*know_GE*_*i*_) was used to test our hypothesis that the impact of knowledge on the acceptance of new technology varies at different stages of diffusion. Specifically, we expect information to become more impactful in the later stages of diffusion. The variable *know_GE* represents the score derived from responses to seven questions regarding knowledge of GE technology (see Appendix: Table A1). These questions cover the technology itself and its applications in agriculture and medicine and were based on information from the “Bio Station” website.^69^ This website is partly operated by the project “Technologies for Smart Bio-industry and Agriculture” under Japan’s Cross-ministerial Strategic Innovation Promotion Program (SIP).^70^

The vector $X_{i}$ comprises socio demographic variables, such as gender, age, education, and attitudes toward science. Descriptive statistics and definitions for these variables are shown in Table 3. We used *d_fem* as a dummy variable for gender, with *age* represented as a continuous variable ranging from the 20s to the 60s, and *d_child* indicating whether the respondent has children. The dummy variable *d_edu* represents respondents’ educational background, specifically whether they have a college education. Finally, the variable *tec* represents respondents’ interest in science and technology, which we expect to positively impact their evaluations of new technologies.

## 4. Results and Discussion

### 4.1. Results of OLS Regression

#### 4.1.1. Effectiveness of Knowledge

Table 4 presents the results of the multiple regression analysis for the three categories: CONS, PROD, and MED. The annual impact of technological knowledge is represented by the term *d_year×know_GE*_*i*_. The coefficients for the CONS category are positively and statistically significant from 2021 to 2023. The absolute value of the coefficient increased gradually, starting from 0.062 in 2021, rising to 0.110 in 2022, and reaching 0.158 in 2023. This indicates that the effectiveness of knowledge on technology acceptance in the CONS category is positive, with its contribution becoming even more pronounced in recent years.

**Table 4.** Results of OLS regression.

	Dependent variable		
	CONS	PROD	MED
*d_fem*	0.030	−0.190***	0.132***
	(0.019)	(0.020)	(0.020)
*age*	−0.010***	−0.009***	0.001
	(0.001)	(0.001)	(0.001)
*d_child*	0.093***	0.012	0.054***
	(0.019)	(0.02)	(0.020)
*tec*	0.458***	0.328***	0.633***
	(0.017)	(0.017)	(0.018)
*d_edu*	−0.061***	−0.081***	0.004
	(0.019)	(0.019)	(0.019)
*d_2019*	−0.125*	−0.044	−0.040
	(0.071)	(0.074)	(0.066)
*d_2021*	−0.167**	0.067	−0.153**
	(0.074)	(0.077)	(0.069)
*d_2022*	−0.383***	−0.035	−0.459***
	(0.075)	(0.080)	(0.076)
*d_2023*	-0.464***	*-0.085*	-0.475***
	(0.075)	(0.076)	(0.077)
*know_GE*	*-0.034*	0.066***	*-0.021*
	(0.025)	(0.025)	(0.022)
*d_2019× know_GE*	0.039	0.022	−0.012
	(0.031)	(0.033)	(0.028)
*d_2021× know_GE*	0.062*	−0.003	0.021
	(0.034)	(0.036)	(0.030)
*d_2022× know_GE*	0.110***	*0.043*	0.080**
	(0.033)	(0.035)	(0.032)
*d_2023× know_GE*	0.158***	0.088**	0.091***
	(0.033)	(0.034)	(0.031)
Constant	2.218***	2.034***	1.739***
	(0.079)	(0.079)	(0.082)
Observations	6,234	6,234	6,234
Adjusted R2	0.181	0.139	0.272
F Statistic (df = 14; 6219)	99.418***	72.980***	167.264***

*p < .1, **p < .05, ***p < .01.

Similarly, the coefficient for the MED category is also positive and significant for the two-year period of 2022–2023, with its absolute value increasing from 2022 to 2023. This indicates that the effectiveness of knowledge in the MED category is not only positive but has also increased in recent years. Respondents who possessed greater knowledge about GE technology are more likely to support promoting GE technology for both CONS and MED purposes. Consequently, the provision of information has grown increasingly important as technology has diffused.

Previous studies in Canada,^45^ Korea,^23^ and Norway^47^ have shown that knowledge of technology enhances evaluations of GE technology. In Japan, knowledge plays a crucial role in increasing acceptance of GM vaccine rice.^64^ Beghin and Gustafson^28^ reviewed consumer attitudes toward novel foods produced using GE technology and highlighted that higher levels of knowledge about science and technology promote acceptance of these products.

Knowledge is believed to positively influence consumer acceptance by affecting their perceived risks and benefits. Consumers often harbor vague anxieties and fears about new technologies.^71^ However, understanding specific risks and their management can empower consumers to make more informed decisions about GE products. Additionally, knowledge is essential for addressing consumers’ biased perceptions of GE products. When individuals lack sufficient knowledge to anticipate or mitigate potential negative impacts, they tend to worry about unfamiliar risks.^72,73^ Therefore, enhancing consumers’ understanding of GE technology can potentially foster more positive attitudes toward it.

In this study, we demonstrate that knowledge can enhance consumer acceptance and confirm that its impact is greater in the later stages of technology diffusion. This aligns with the findings of van Giesen et al.,^39^ who noted that cognitive responses become more significant in the later stages of nano-technology diffusion.

Interestingly, the interaction term between knowledge and year became significant in the following order: CONS, MED, and PROD, as the survey year progressed. In the PROD category, 2023 was the only year that had a positive and significant effect on public acceptance. This suggests that knowledge of GE technology positively influences respondents’ motivation to promote these technologies, although the timing varies slightly by category. Knowledge promotes product development, which directly benefits customers and is intuitively understood, particularly in the CONS and MED categories during the early stages of diffusion. In contrast, the indirect nature of benefits associated with PROD products tends to lag behind the other two categories, as these products mainly benefit producers, such as farmers or fishermen. This aligns with findings from previous studies in Norway.^47^ However, the impact of knowledge on promoting PROD in 2023 is 0.154 (with *know_GE* contributing 0.066 and *d_2023×know_GE* contributing 0.088), making it the largest among the three categories. Knowledgeable consumers increasingly appreciate GE products in the PROD category, particularly in the later stages of the diffusion process.

While products in the PROD category may be less appealing to consumers, these technologies are crucial for enhancing agricultural productivity. The occurrence of food-related incidents has heightened public interest in food production and production technology, including breeding methods. To address this growing public interest, especially in the PROD category, it is essential that information is readily accessible and that the content is regularly updated to encourage positive consumer behaviors.

#### 4.1.2. Yearly Differences

We examined how the intention to promote GE products varies by year while controlling for other variables. In the CONS category, the coefficient for the year dummy was negative and statistically significant, with absolute values gradually increasing from 0.125 in 2019 to 0.464 in 2023. This indicates that public intention to promote GE technology in the CONS category has shifted increasingly toward opposition. Research shows that Chinese consumers perceived GM food negatively during the COVID-19 period from 2019 to 2020.^65^ Similar trends are anticipated in Japan, potentially continuing until 2023.

The year dummies for 2021 to 2023 in the MED category are also negative and significant, with absolute values of the coefficients gradually increasing. In November 2018, the case of the genome-edited twin babies in China faced strong condemnation from both the international and scientific communities.^74^ This news was also reported in Japan, leading to ethical concerns that may have diminished the acceptance of GE technology for medical applications (MED), at least from 2018 to 2019. Although a survey conducted in March 2021 indicated a slight recovery in acceptance, there was a significant decline in acceptance observed in 2022 and 2023.

**Figure 5.**
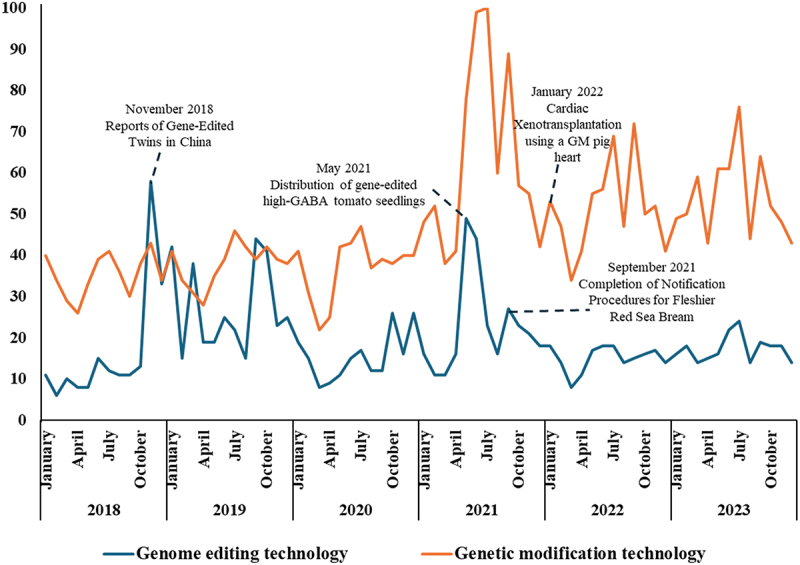
Search volume for the terms “genome editing technology” and “genetic modification technology” in Japanese (google).

This significant decline warrants explanation. Although we do not have clear and specific reasons, two possible explanations emerge. The first involves sensational news regarding genetically modified pig kidneys that were transplanted into brain-dead patients in the U.S.^75^ Although the technology used was genetic modification rather than gene editing, consumers may have sought related information (see Fig. 5). The second reason pertains to food-related incidents. In extraordinary circumstances, such as the COVID-19 pandemic and the Russia-Ukraine conflict, the perceived importance of medical benefits may diminish while interest in food and agriculture increases. Concerns about food safety and security have risen during the COVID-19 pandemic^76^ and the Russia-Ukraine conflict, which may also impact public acceptance of GE technology in the MED field. As consumers become more concerned about food and food prices, they may exhibit less interest in advancements in medical technology.

### 4.2. Respondent Characteristics

Finally, we assessed the impact of various respondent characteristics on public intentions. The coefficients for the female dummy variable are positive for the CONS and MED categories but negative and significant for PROD. This indicates that women tend to hold more positive views of CONS and MED while expressing more negative opinions about PROD compared to men. Previous studies in the U.S.^26,43^ have indicated that women generally hold more negative opinions than men. Our results further suggest that these views vary depending on the purpose of the technology.

The age coefficient is negative and significant for the CONS and PROD categories, suggesting that older individuals tend more likely to hold negative opinions about CONS and PROD development compared to younger generations. This aligns with previous reviews showing that older adults are more likely to perceive the development of new technologies negatively than younger individuals.^26^ The coefficient for dummy variable representing respondents with children (*d_child*) is positive and significant in the CONS and MED categories, suggesting that parents with children are more likely to perceive GE technology positively than those without children. Parents tend to value the long-term benefits of technology, especially its applications in the MED category.

The dummy variable *d_edu* is found to be negative and significant for both the CONS and PROD categories. This finding somewhat contradicts our earlier results regarding knowledge. However, higher levels of education encompass various types of knowledge across different fields. Educated respondents do not uniformly support all GE technologies; their support varies depending on the specific field. In contrast, the coefficient for the variable *tec* is positive and significant across all three categories. Respondents with a greater interest in science and technology tended to exhibit stronger positive opinions regarding technology adoption. Consumers form their attitudes by weighing perceived benefits against perceived risks.^27^ This study demonstrates that a heightened interest in science and technology can diminish the perceived risks associated with GE technology.

## 5. Conclusion

The demand for GE technology has been increasing due to the growing global population, the need for climate change countermeasures, and the demand for new medical technologies. Despite the potential benefits of GE technology in addressing food and medical-related challenges, societal agreement on its use remains elusive. In the Japanese market, while GE products have already been introduced, consumer responses are not yet well understood.

The purpose of this study was to investigate public responses to GE technology across different applied categories over multiple years, with a specific focus on understanding the impact of knowledge on technology acceptance. By examining these factors, we aimed to analyze whether public acceptance varies by category and stage of technology diffusion. Our survey, conducted from 2018 to 2023, encompassed various categories, including agricultural products and medical applications.

Our results from the multiple regression analysis revealed that the influence of knowledge on GE technology acceptance has strengthened over time. Although the importance of information has been established in numerous prior studies, this study highlights that its impact varies according to the diffusion stage. The observed increase in the influence of knowledge in the later years of the study underscores the necessity of continuously providing accurate information to accelerate the adoption of technology within society. In other words, the public continues to seek correct information, and it is essential for society to remain informed.

The regulatory and technological environments surrounding GE technology are in constant flux. As advancements in GE technology continue to emerge and regulatory frameworks evolve, public acceptance of these technologies is likely to shift as well. Transparent communication regarding the benefits and potential risks of new technologies may enhance consumers’ familiarity with their applications and gradually alter their attitudes.^77^ In Japan, GE technology has been introduced into the market more smoothly than GM technology; however, public evaluations of GE technology may still change as it becomes more widely adopted. Therefore, it is essential to keep the public informed even after commercialization, enabling them to develop a deeper understanding and to make decisions based on sufficient information and knowledge.

Policymakers and developers must continue their efforts to provide accessible and updated information to the public. These outreach activities are especially effective during the diffusion of technology. Furthermore, it is crucial to adopt varied approaches in outreach and information dissemination tailored to different consumer groups. For instance, older individuals generally view GE technology more negatively than younger generations. However, older adults with children may become less critical or even more supportive when presented with information related to the CONS category, such as improved taste or enhanced health benefits, or the MED category, such as new treatment methods. Thus, information should be tailored to the specific needs and characteristics of the target audience in outreach activities. These findings can also be applied to new technologies that may emerge as advancements in science and technology progress.

Although the present study reveals important findings, it has several limitations. First, we have not clarified how public knowledge interacts with other individual attributes. While this study has produced significant findings regarding the changing role of knowledge at different stages of diffusion – based on the interaction between knowledge and the year of diffusion – knowledge also mutually influences individuals’ personal attributes. To provide more effective information to specific target groups, it is crucial to elucidate this relationship in future research.

The second limitation pertains to the geographic scope. The differences in acceptance by purpose observed in this study align with results from an international comparison involving five countries in Europe and North America, where the application of medical technology for HIV treatment was evaluated more favorably than the disease resistance of agricultural products such as wheat and pigs.^48^ Furthermore, the importance of information dissemination in increasing social acceptance is consistent with previous studies from China^38^ and South Korea,^23^ both of which are part of East Asia. The survey was conducted online, targeting Japanese participants. Japan is an unique region where GM crops faced strong opposition, yet the introduction of GE products has been successful.^18^ Public evaluations of GE technology are known to vary by country or region.^21,44,48^ To further generalize these findings, comparative studies with other countries and regions will be necessary.

## Supplementary Material

Supplement information.docx
