# Supplementary material for: Growing knowledge impact of gene-editing technology on public acceptance: a longitudinal analysis in Japan
Source: GM Crops Food. 2024 Dec 6;15(1):411–28. doi: 10.1080/21645698.2024.2435709 (PMC11633139; doi:10.1080/21645698.2024.2435709)
Supplement: Supplement information.docx [file KGMC_A_2435709_SM1596.docx]

Table A1. Definition and descriptive Statistics of variable, *know_GE*

(1) Definitions of *know_GE*

| How familiar were you with each of the following statements regarding genome editing technology? Please answer before answering this questionnaire. | | 1. Nothing at all，  2. Not much  3. Have heard of it  4. Know some information  5. Very knowledgeable |
| --- | --- | --- |
| know_GE_1 | Genome editing technology is a technology that controls the function of specific genes | |
| know_GE_2 | Genome editing technology can greatly increase the speed of breeding | |
| know_GE_3 | Genome editing technology can theoretically be applied to almost all plants and animals | |
| know_GE_4 | Genetic modification by genome editing technology introduces the same mutations as those in nature. | |
| know_GE_5 | It is technically difficult to distinguish gene modifications by genome editing technology from natural mutations | |
| know_GE_6 | Genome editing technology is expected to enable the treatment of diseases | |
| know_GE_7 | Genome editing technology can modify the target mutation more precisely than conventional mutation induction | |

Table A1. Definition and descriptive Statistics of variable, *know_GE*(continued)

(2) Descriptive Statistics for each *know_GE*

|  | 2018 | 2019 | 2021 | 2022 | 2023 |
| --- | --- | --- | --- | --- | --- |
| know_GE_1 | 2.163  (1.047) | 2.363  (1.050) | 2.348  (1.064) | 2.319  (1.114) | 2.344  (1.116) |
| know_GE_2 | 2.106  (1.068) | 2.257  (1.056) | 2.250  (1.052) | 2.297  (1.096) | 2.278  (1.080) |
| know_GE_3 | 2.039  (1.043) | 2.244  (1.089) | 2.104  (1.037) | 2.181  (1.068) | 2.219  (1.094) |
| know_GE_4 | 1.965  (1.009) | 2.088  (1.027) | 1.979  (0.997) | 2.112  (1.081) | 2.146  (1.066) |
| know_GE_5 | 1.833  (0.933) | 1.972  (0.974) | 1.880  (0.961) | 2.013  (1.011) | 2.063  (1.030) |
| know_GE_6 | 2.242  (1.139) | 2.421  (1.134) | 2.381  (1.154) | 2.370  (1.158) | 2.408  (1.130) |
| know_GE_7 | 1.940  (1.011) | 2.122  (1.050) | 2.077  (1.052) | 2.139  (1.063) | 2.195  (1.085) |

Note: The upper row indicates mean values and the numbers in parentheses are standard deviations.

Table A2. Definition and descriptive Statistics of variable, *tec*

(1) Definitions of *tec*

| We would like to ask you about science and technology development in Japan. To what extent do you think each of the following technological developments should be promoted? Please choose the one that comes closest to your opinion. | | 1: Do not promote  2: Should not promote  3: Cannot decide  4: Should promote  5: Promote |
| --- | --- | --- |
| tec_1 | Earthquake resistance and disaster prevention | |
| tec_2 | Biotechnology in food production (i.e., genome editing/genetic engineering) | |
| tec_3 | Regenerative organ medicine | |
| tec_4 | Robotic technology | |
| tec_5 | Climate change simulations | |
| tec_6 | Driver safety | |
| tec_7 | AI technology applications | |
| tec_8 | Less nature-dependent technology of fishery production | |
| tec_9 | Technology takeover | |
| tec_10 | Medical application for genetic information | |
| tec_11 | Space development | |
| tec_12 | Ocean development | |

Table A2. Definition and descriptive Statistics of variable, *tec* (continued)

(2) Descriptive statistics for each *tec*

|  | 2018 | 2019 | 2021 | 2022 | 2023 |
| --- | --- | --- | --- | --- | --- |
| tec_1 | 4.136  (0.834) | 4.170  (0.838) | 4.252  (0.791) | 4.138  (0.865) | 4.109  (0.875) |
| tec_2 | 3.431  (0.894) | 3.453  (0.943) | 3.527  (0.953) | 3.563  (0.953) | 3.642  (0.952) |
| tec_3 | 3.931  (0.931) | 4.010  (0.884) | 4.048  (0.881) | 3.985  (0.914) | 3.932  (0.918) |
| tec_4 | 3.448  (0.943) | 3.548  (0.908) | 3.644  (0.925) | 3.668  (0.940) | 3.671  (0.909) |
| tec_5 | 3.688  (0.861) | 3.677  (0.855) | 3.881  (0.840) | 3.859  (0.873) | 3.775  (0.900) |
| tec_6 | 3.909  (0.968) | 3.948  (0.895) | 4.014  (0.958) | 3.984  (0.934) | 3.934  (0.951) |
| tec_7 | 3.496  (0.958) | 3.557  (0.923) | 3.710  (0.909) | 3.703  (0.944) | 3.711  (0.929) |
| tec_8 | 3.827  (0.889) | 3.848  (0.851) | 3.950  (0.864) | 3.890  (0.912) | 3.919  (0.887) |
| tec_9 | 3.999  (0.887) | 3.906  (0.880) | 4.063  (0.903) | 3.941  (0.890) | 3.917  (0.915) |
| tec_10 | 3.531  (0.893) | 3.555  (0.907) | 3.694  (0.914) | 3.699  (0.926) | 3.659  (0.929) |
| tec_11 | 3.305  (0.928) | 3.306  (0.931) | 3.436  (0.963) | 3.446  (0.949) | 3.452  (0.968) |
| tec_12 | 3.737  (0.882) | 3.754  (0.902) | 3.894  (0.906) | 3.777  (0.916) | 3.810  (0.886) |

Note: The upper row indicates mean values and the numbers in parentheses are standard deviations.

Table A.3. Items classified into three categories

| Development objectives | Items |
| --- | --- |
| Consumer benefits | 1. Tomatoes and melons with high sugar content |
|  | 2. Potatoes that do not produce solanine |
|  | 3. Decaffeinated tea |
|  | 4. Apples that do not turn brown |
|  | 5. Delicious rice |
|  | 6. Feed rice that is high in amino acids |
|  | 7. Allergy-free buckwheat |
|  | 8. Vegetables effective in treating dementia |
|  | 9. Rice effective in treating hay fever |
| Producer benefits | 1. Flowers with unusual colors and shapes |
|  | 2. Apples with fast breeding speed |
|  | 3. Corn, forage, and rapeseed with herbicide resistance |
|  | 4. Wheat with herbicide resistance |
|  | 5. Oilseed soybeans with resistance to pests |
|  | 6. Rice and papaya with disease resistance |
|  | 7. Tuna with high aquaculture efficiency |
|  | 8. Pigs with increased meat production |
|  | 9. Cattle without horns |
| Medical technology | 1. Treatment of muscular dystrophy |
|  | 2. Prevention of AIDS onset in patients |
|  | 3. Treatment of Parkinson's disease |
|  | 4. Treatment of cancer |
|  | 5. Prevention of hereditary diseases |
|  | 6. Recovery from liver disease |
